# Supplementary material for: hENT1 Predicts Benefit from Gemcitabine in Pancreatic Cancer but Only with Low CDA mRNA
Source: Cancers (Basel). 2021 Nov 17;13(22):5758. doi: 10.3390/cancers13225758 (PMC8616255; doi:10.3390/cancers13225758)
Supplement: Supplementary file 1 [file cancers-13-05758-s001.zip › cancers-1418213-supplementary.pdf]

SUPPLEMENTARY METHODS

The *RNAscope SpotStudio® v 1.0* technology builds on the *Definiens®* software's standardised detection of individual cells, and allows detection and enumeration of DAB spots and spot clusters and hence transcript expression. First, four parameters setting the thresholds for identifying individual cells and spots were optimised: haematoxylin intensity, nuclear diameter, DAB spot stain intensity, and spot diameter, before automated enumeration occurred. Using the positive (PPIB) and negative (DapB) control slides the optimum parameter settings for this analysis were determined based on maximising spot detection whilst minimising background. Two separate set-ups were compared (see Table S1), chosen based on principles stated by the software supplier ACD (http://www.acdbio.com). Both set-ups were subsequently quality assessed on all 3 markers: PPIB, CDA and hENT1. Tissue was analysed for: mean number of spots per cell (with spot clusters included), mean number of single spots per cell (with spot clusters excluded), and proportion of cells displaying various numbers of spots (Group 1 = 0 spots per cell, Groups 2 = 1-5 spots per cell, and Group 3 = ≥ 6 spots per cell).

Quality assessment of the *SpotStudio®* platform: comparing the outcomes of two different software setups.

A subset of TMA blocks (25%) were randomly chosen and analysed with both setups for PPIB, CDA and hENT1. The outcome data was presented as 'average number of estimated spots per cell' and the correlation between the outcomes of the two different setups was assessed. Figure S1 depicts the correlation of all individual samples analysed for the respective marker and settings.

The outcome data was consistent for both parameter settings, with Pearson's correlation coefficients ranging between 0.80 - 0.95 for all three markers investigated. This suggests data generated with analysis by *RNAscope SpotStudio®* are robust and reproducible for both parameter threshold settings. Analysis 1 settings were used for all subsequent data analysis.

Immunohistochemistry staining of tissue microarrays with CDA antibody.

Immunohistochemistry (IHC) was performed on ESPAC-3(v2) formalin-fixed, paraffin-embedded tissue microarrays sectioned at 4 μm thickness and placed onto SuperFrost Ultra Plus™ adhesion slides (Fisher Scientific, Leicestershire, UK). Antigen retrieval was performed using the Dako PT-Link and low pH antigen retrieval solution (Dako UK Ltd, Cambridgeshire, UK). Subsequently IHC was performed using Dako EnVision kit according to manufacturer's instructions. Primary CDA antibody (ab137605, Abcam, Cambridgeshire, UK) was used at a previously optimised concentration (1:400). Sections were then counterstained with hematoxylin.

Quantification using *Definiens*® software

CDA antibody staining was verified by a pathologist (FC) for specificity and staining was localised mainly to the cytoplasm with some cells displaying membrane stain. A range of staining intensity was observed across different cores as shown in Figure S2. Slides were scanned using Aperio image capture device (Leica Biosystems) and Definiens software (Definiens, Munich Germany) was used to quantify the intensity of CDA staining in the tumour cells using a computational approach to machine learning. The average marker intensity score was used as a measure of CDA staining in the tumour cells. Discrepancies in TMA section staining were controlled for using box-plots of each TMA score set to ensure outlying TMAs were removed from further analysis. Results from the IHC scoring were matched with results from RNAscope for the same patients and correlations calculated (Figure S3).

SUPPLEMENTARY TABLES

Table S1. Parameter settings for each analysis to determine optimum spot detection and scoring.

	Hematoxylin intensity (AU)	Nuclear diameter (μm)	Spot staining intensity (AU)	Spot diameter (μm)
Analysis 1	0.10	8.5	0.16	1.5
Analysis 2	0.19	7.5	0.15	1.0

Table S2. Cox model fit for survival hazard using different methods of determining CDA mRNA expression showing -2log(likelihood) for both 5-FU and gemcitabine treated patients. There was very little difference in likelihood using the different scoring methods. MSPC = mean number of single spots per cell (excluding clusters), SPC = mean number of spots per cell (including clusters), PCTS = percentage of cells with spots, H-score = mean spots per cell × (0 × Group 1 + 1 × Group 2 + 2 × Group 3)/100 where Group 1 = 0 spots per cell, Group 2 = 1-5 spots per cell, and Group 3 ≥ 6 or more spots per cell.

		**Scoring Method**			
		**MSPC**	**SPC**	**PCTS**	**Hscore**
**5-FU**	P-value	**0.034**	0.109	0.098	0.084
	-2log(likelihood)	**762.3**	764.0	763.6	763.9
**GEM**	P-value	0.221	0.184	0.166	0.275
	-2log(likelihood)	930.7	930.5	930.3	931.1

Table S3. Relationship between CDA mRNA expression and clinical and pathological factors in the 277 chemotherapy treated patients.

Characteristic		Number	CDA MSPC mean (sd)	*P-value*
**Resection Margin**	Negative	156	0.47 (0.23)	0.486
	Positive	121	0.47 (0.22)	
**Lymph Node Status**	Negative	58	0.47 (0.23)	0.532
	Positive	219	0.47 (0.23)	
**Tumor stage**	1	16	0.44 (0.14)	**0.048**
	2	68	0.46 (0.23)	
	3	182	0.48 (0.24)	
	4	9	0.47 (0.16)	
**Tumor grade**	Well	17	0.50 (0.23)	0.352
	Moderate	187	0.46 (0.24)	
	Poor	67	0.51 (0.24)	
**Local invasion**	No	145	0.47 (0.22)	0.406
	Yes	129	0.47 (0.23)	
**Maximum Tumor Diameter**	<30mm	127	0.47 (0.22)	0.538
	≥30mm	140	0.47 (0.24)	
**Diabetes mellitus**	No	208	0.47 (0.23)	0.619
	Yes	62	0.48 (0.23)	
**Gender**	Male	163	0.49 (0.24)	0.920
	Female	114	0.45 (0.21)	
**Age (Years)**	<64	132	0.46 (0.22)	0.770
	≥64	145	0.48 (0.24)	

SUPPLEMENTARY FIGURES

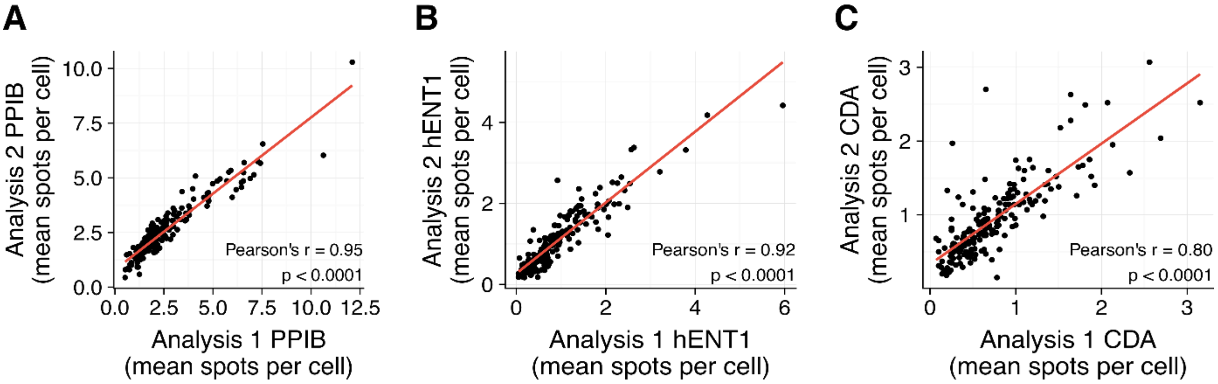

Figure S1 Comparison of Analysis 1 vs Analysis 2 SpotStudio® configurations. The settings for Analysis 1 and 2 are given in Table S1. Pearson's correlation coefficients were calculated for each comparison, measuring MSPC scores of **A:** PPIB (n = 200), **B:** hENT1 (n = 194), and **C:** CDA (n = 192).

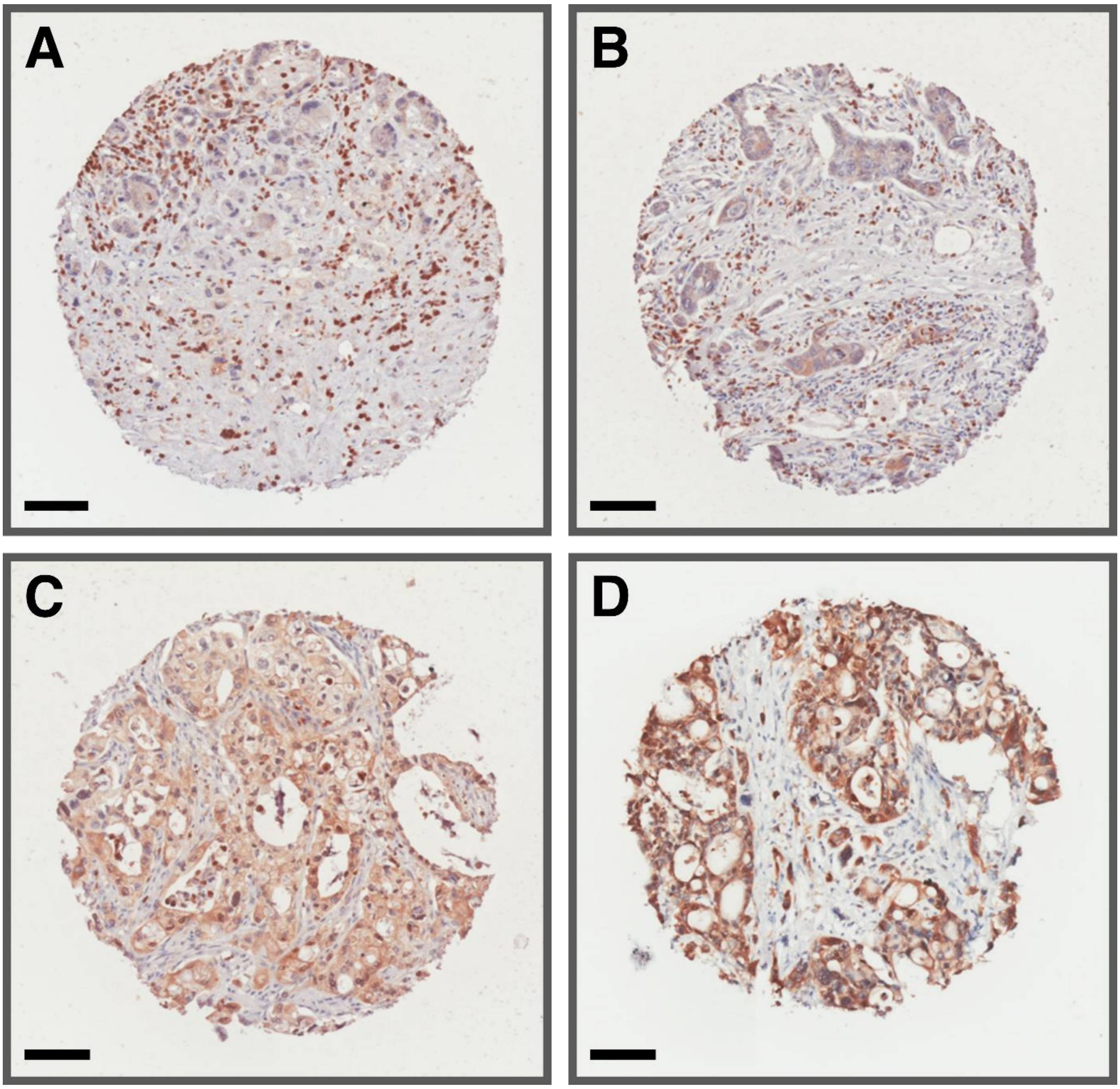

Figure S2 Examples of the range of CDA IHC staining intensity across different cores. **A:** Negative staining with positive inflammatory cells; **B:** Weak staining; **C:** Moderate staining; **D:** Strong staining. Scale bar = 100μm.

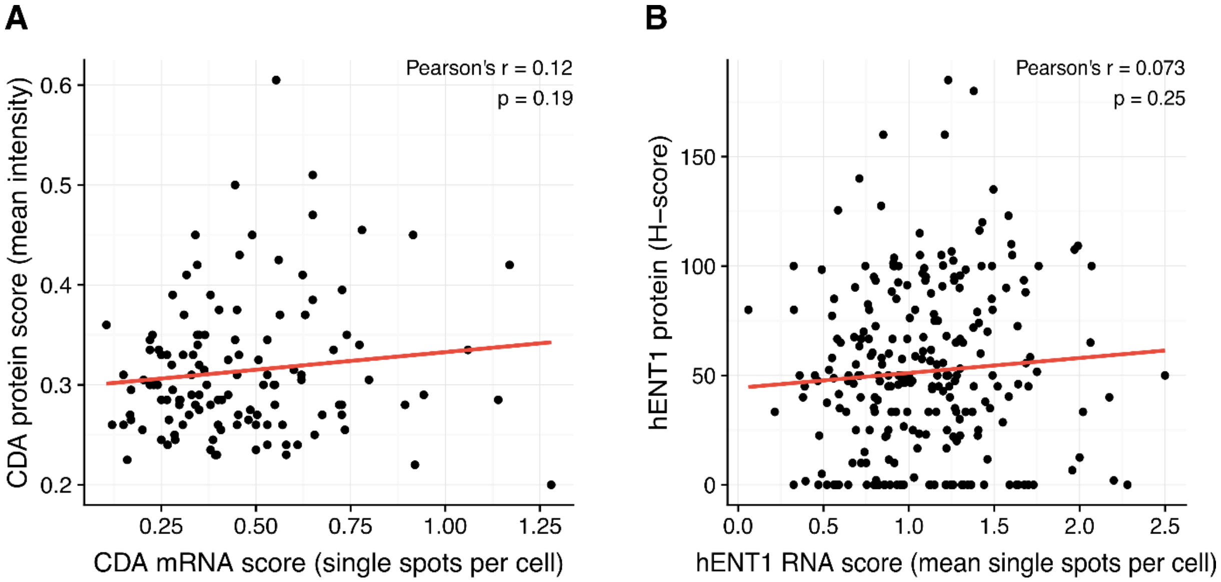

Figure S3 Comparison of CDA and hENT1 mRNA expression with protein levels. **A:** Correlation between CDA mRNA expression level (MSPC) and CDA protein expression (average marker intensity). **B:** Correlation between hENT1 mRNA expression level (MSPC) and hENT1 protein expression (H-score). Pearson's correlation coefficient was calculated for each comparison.

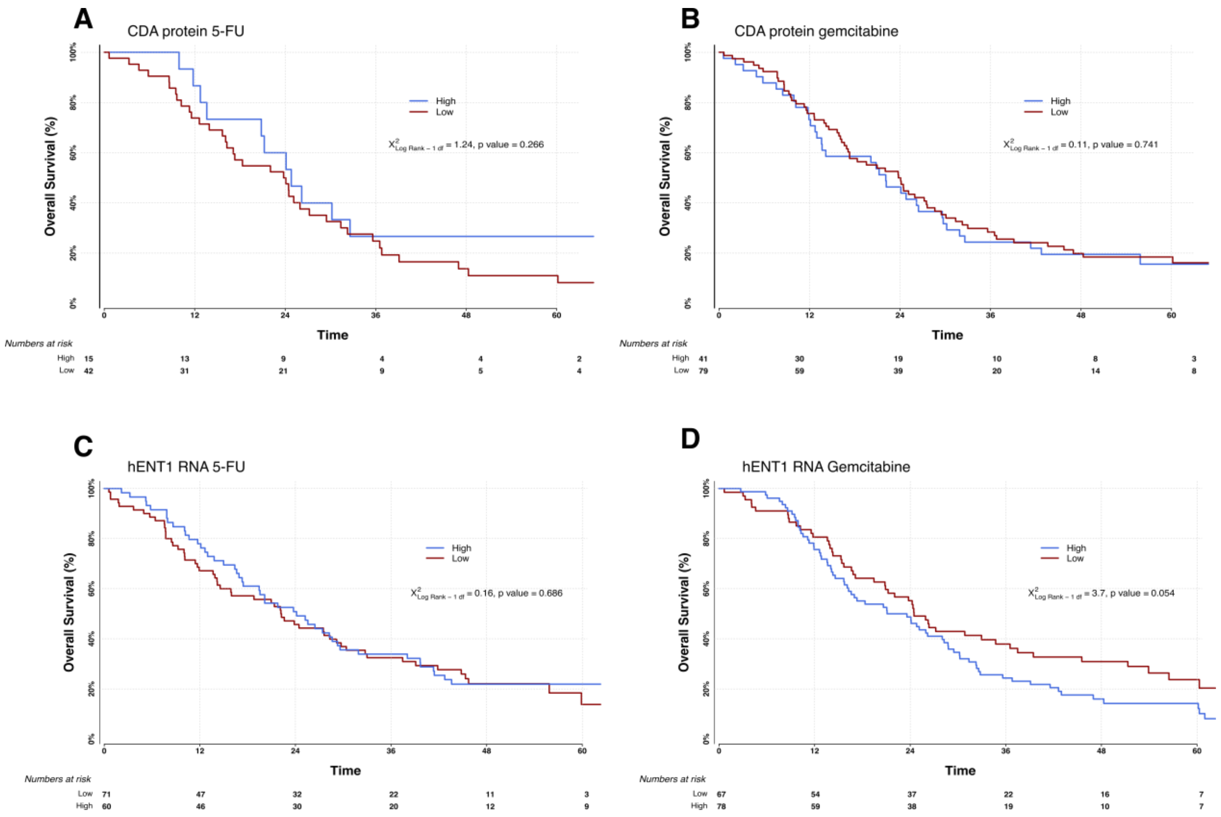

Figure S4 Kaplan-Meier survival curves for analyses of CDA protein and hENT1 mRNA alone. Graphs show response to adjuvant chemotherapy in patients with high or low CDA protein expression. In the 5-FU (**A**) or gemcitabine (**B)** arms, or the effect of high or low hENT1 mRNA expression in response to 5-FU (**C**) or gemcitabine **(D)**. All groups and the number of at risk individuals are shown in each graph. All statistical tests were log-rank analyses using two-sided χ^2 tests.

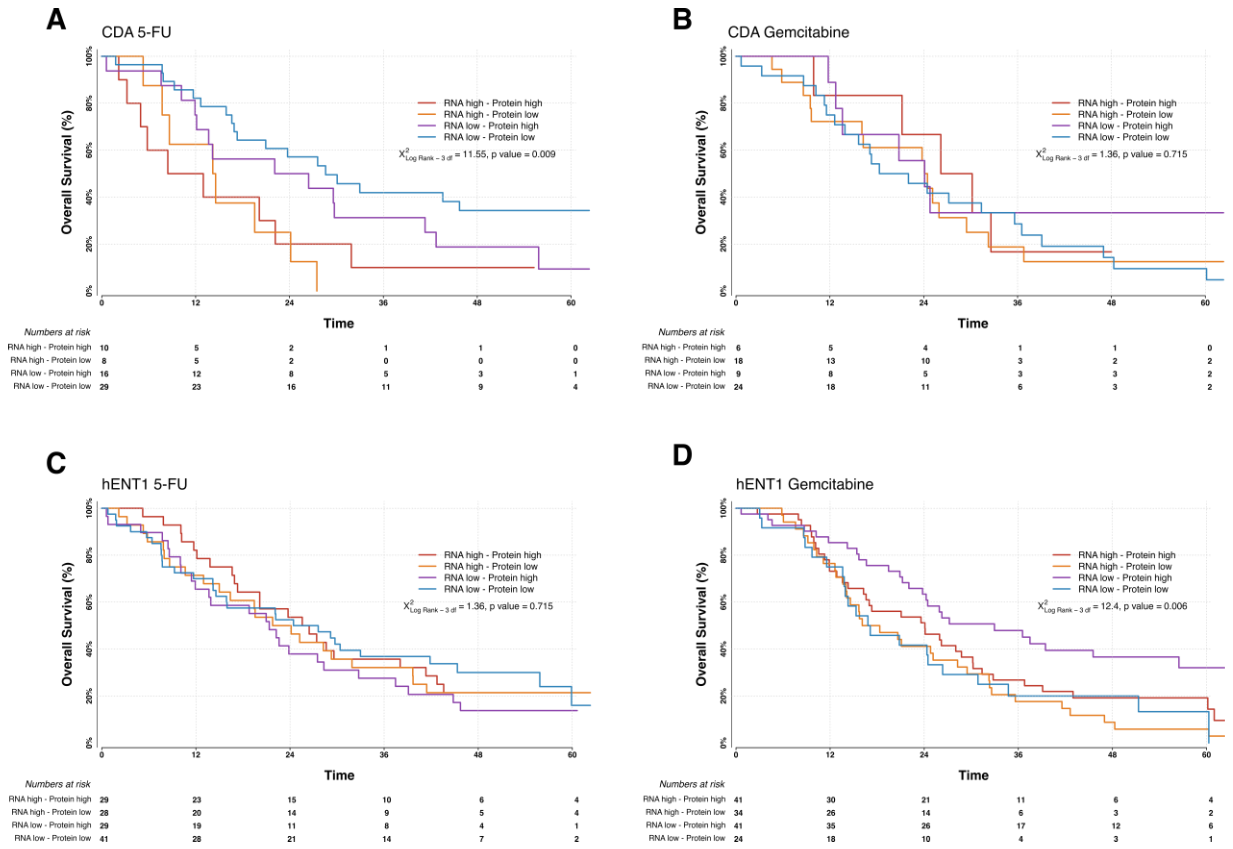

Figure S5 Kaplan-Meier survival curves for analyses of combined CDA mRNA with CDA protein or combined hENT1 protein with hENT1 mRNA. Graphs show response to treatment with 5-FU (**A** and **C)** and gemcitabine (**B** and **D**). All groups and the number of at risk individuals are shown for each graph. All p-values were determined by log-rank analyses using two-sided χ^2 tests. The only significant differences in survival were due to CDA mRNA (**A**) and hENT1 protein (**B**).
